# Supplementary material for: The Quality of Life in Patients with Epilepsy in the Context of Sleep Problems and Sexual Satisfaction
Source: Brain Sci. 2021 Jun 11;11(6):778. doi: 10.3390/brainsci11060778 (PMC8231274; doi:10.3390/brainsci11060778)
Supplement: Supplementary file 1 [file brainsci-11-00778-s001.zip › brainsci-1234489-supplementary.pdf]

Place........................ Date...................

PART I: FOR THE PHYSICIAN

The questionnaire contains information concerning the patient and his/her disease. We kindly ask you to complete BOTH PAGES of the questionnaire.

Patient's initials............................... **Age** ..... **Sex** M ❑ F ❑

How many years has the patient had epilepsy? ....
The etiology of epilepsy ....

1. Current medications (list all preparations, please)

....

………………………………………………………………………………………..

2. Is epilepsy resistant to pharmacological treatment? Yes ❑ No ❑

3. Has the patient experienced a seizure since the last visit?

Yes ❑ How many? .... No ❑
type of seizure (specify) ....

4. TYPE AND FREQUENCY OF EPILEPTIC SEIZURES

a) generalized tonic-clonic seizures ❑

no seizures over the past 6 months ❑
1 - 2 seizures over the past 6 months ❑
3 - 5 seizures over the past 6 months ❑
1 or more seizures per month ❑
1 or more seizures per week ❑
1 or more seizures per day ❑

b) complex partial seizures ❑

no seizures over the past 6 months ❑
1 - 2 seizures over the past 6 months ❑
3 - 5 seizures over the past 6 months ❑
1 or more seizures per month ❑
1 or more seizures per week ❑

1 or more seizures per day ❑

c) simple partial seizures ❑

no seizures over the past 6 months ❑
1 - 2 seizures over the past 6 months ❑
3 - 5 seizures over the past 6 months ❑
1 or more seizures per month ❑
1 or more seizures per week ❑
1 or more seizures per day ❑

d) absence seizures ❑

no seizures over the past 6 months ❑
1 - 2 seizures over the past 6 months ❑
3 - 5 seizures over the past 6 months ❑
1 or more seizures per month ❑
1 or more seizures per week ❑
1 or more seizures per day ❑

e) myoclonic seizures ❑

no seizures over the past 6 months ❑
1 - 2 seizures over the past 6 months ❑
3 - 5 seizures over the past 6 months ❑
1 or more seizures per month ❑
1 or more seizures per week ❑
1 or more seizures per day ❑

f) unclassified ❑

no seizures over the past 6 months ❑
1 - 2 seizures over the past 6 months ❑
3 - 5 seizures over the past 6 months ❑
1 or more seizures per month ❑
1 or more seizures per week ❑
1 or more seizures per day ❑

PART II: FOR THE PATIENT

Below you will find questions concerning your disease and its influence on your life. We kindly ask you to complete them. Please, read each question carefully and mark the answer which is the most consistent with your opinion. **There are no correct or incorrect, better or worse answers.** We only want to find out what your opinion is on the issues included in the questions below.

Full name (or initials)…………

5. Marital status and family

Married ❑
Unmarried ❑
Divorced / separated ❑
Widowed ❑
Domestic partnership ❑

CHILDREN number (total) ..... financially dependent .....

6. Education

Tertiary ❑
Secondary ❑
Vocational ❑
Primary ❑

7. Place of residence

Village ❑
Town <10,000 inhabitants ❑
Big town >10,000<100,000 inhabitants ❑
City <100,000 inhabitants ❑

8. Occupation

a. full-time job ❑
b. odd job ❑
c. retirement pensioner ❑
b. disability benefit ❑
c. disability benefit + (+job or +retirement pension) ❑
d. financially dependent on the family ❑
e. student/pupil ❑

continued on the following page

9. How often do you visit a neurologist?

less often than once a year	❑
once a year	❑
2-3 times a year	❑
4-6 times a year	❑

10. Do you feel supported and accepted by the family?

I=========I=========I=========I=========I========I=========I
Always Very often Often Sometimes Seldom Very seldom Never

11. Do you assess your financial situation as satisfactory?

I=========I=========I=========I=========I========I=========I
Always Very often Often Sometimes Seldom Very seldom Never

12. Has anything dramatic happened in your life over the past 6 months which might significantly lower your mood? (death in the family, loss of work, etc.)

I=========I=========I=========I=========I========I=========I
Always Very often Often Sometimes Seldom Very seldom Never

13. Do you have a feeling that others feel uncomfortable in your company <u>because of epilepsy</u>?

I=========I=========I=========I=========I========I=========I
Always Very often Often Sometimes Seldom Very seldom Never

14. Do you have a feeling that you are not promoted at work <u>because of epilepsy</u>?

I=========I=========I=========I=========I========I=========I
Always Very often Often Sometimes Seldom Very seldom Never

15. Have you ever had to resign from a job or lost a job <u>because of epilepsy</u>?

I=========I=========I=========I=========I========I=========I
Always Very often Often Sometimes Seldom Very seldom Never

16. Do you feel ashamed and embarrassed <u>because of epilepsy</u>?

I=========I=========I=========I=========I========I=========I
Always Very often Often Sometimes Seldom Very seldom Never

continued on the following page

17. Do you have problems with falling asleep?

I=========I=========I=========I=========I========I=========I
Always Very often Often Sometimes Seldom Very seldom Never

18. Are you satisfied with your sex life?

I========I======I========I======I======I=========I=========I
Always Very often Often Sometimes Seldom Very seldom Never Does not apply

19. Do you think epilepsy contributes to problems with sex life?

I=========I=========I=========I=========I========I=========I
Always Very often Often Sometimes Seldom Very seldom Never

20. If so, what problems are they?…………………………………………………..

………………………………………………………………………………………

……………………………………………………………………………………….

We would like to thank you for completing this questionnaire carefully and reliably. The collected data will be used for distinguishing factors associated with the quality of life of persons with epilepsy, which will be the subject of doctoral dissertations.
